# Anaphylaxis to *Moringa oleifera* in North Africa: A case report and review of the literature

**DOI:** 10.1002/ccr3.6193

**Published:** 2022-08-03

**Authors:** Bannour Ichrak

**Affiliations:** ^1^ Department of immuno‐oncology Faculty of medecine of Monastir Laboratory of immunology Fattouma Bourguiba Hospital Monastir Monastir Tunisia

**Keywords:** anaphylaxis, angioedema, *Moringa oleifera*

## Abstract

We describe here the third reported case of anaphylaxis after ingestion of some leavers of *Moringa oleifera*, causing a widespread angioedema, a respiratory distress, and an elevation of serum tryptase. *M. oleifera* leaves were confirmed as the causative allergen by prick testing with fresh leavers.

## Graphical abstract

*Moringa oleifera* is widely consumed, both as a vegetable and in herbal medicines. Unfortunately, it could be responsible for of anaphylaxis reactions despite its anti‐allergic properties.

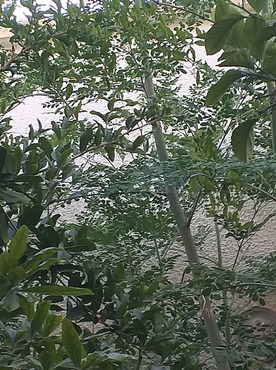

## 1 INTRODUCTION

*Moringa oleifera* (*M. oleifera*) is a commonly used plant cultivated in the tropical and subtropical^1^, ^2^, ^3^ continents and known for its efficacy in combatting severe malnutrition. The leaves, stem, and seedpod of *M. oleifera* can all be eaten. The plant is known for its multiple nutritional and medicinal values.^4^ *M. oleifera* has been reported to contain seven times more vitamin C than oranges, four times more vitamin A than carrots, four times more calcium than milk, two times more protein than yogurt, and three times more potassium than bananas.^5^ It has a high total antioxidant capacity and could be useful for diseases such as cancer, hypertension, and hyperglycemia. It has, in addition, high content of flavonoids, which is associated with its anti‐allergic activity via inhibition of histamine and IL‐4 release that rapidly led the cosmetic industry to incorporate *Moringa* as a compound in various products as moisturizers or skin ointment.^6^ However, we describe here the third case of a life‐threatening anaphylaxis after ingestion of young leaves of *M. oleifera*.

## 2 CASE DETAILS

A 50‐year‐old obese Tunisian teacher with no past medical and no known food or drug allergies presented to the emergency department of « The Excellence Private Hospital in Mahdia from Tunisia » with acute‐onset shortness of breath and a widespread angioedema over his face and body. On initial examination, the patient was in respiratory distress, unable to speak, with diffuse erythema and associated severe pruritus. She described an acute onset of these symptoms approximately 10 min after the ingestion of some young leaves of *M. oleifera*. She had eaten this vegetable one time in her past without symptoms. This plant was brought from Cote d'Ivoire and planted in the Tunisian climatic conditions. The patient was hypotensive, her initial blood pressure was 80/30 ml of mercury (mmHg), pulse rate (HR) 180 beats per minute (bpm), respiratory rate (RR) 33 breaths per minute, oxygen saturation 91% on room air, and body temperature 37°C. She was in acute respiratory distress. Chest examination revealed a wheezing sound. Oropharyngeal examination revealed an edematous soft palate. Her symptoms resolved after injection of adrenaline and parenteral glucocorticoids and antihistamines along with the nebulization of bronchodilators. Serum total IgE was increased at 207 IU/ml. A serum tryptase shortly after admission was elevated at 44, 5 μg/L (reference range 0–12 μg/L), consistent with anaphylaxis. The patient avoided eating *M. oleifera* again. One month later, the serum tryptase level returned to 9 μg/L and skin‐prick tests performed in the emergency department with fresh *M. oleifera* leaves showed positive reactions with a 10 mm wheal diameter, whereas the wheal diameter of positive control (histamine) was 6 mm, and the wheal diameter of negative control (saline) and *Moringa* seed powder were 0 mm (negative control prick tests to both *M. oleifera* seed powder and leaves were observed in four volunteers never exposed to *M. oleifera*).

## 3 DISCUSSION

*Moringa oleifera* is a plant used for medicinal purposes with claimed healing abilities for various inflammatory and chronic diseases.^7^ *Moringa* has shown an inhibitory effect on airway inflammation, and an anti‐asthmatic and anti‐allergic activity via inhibition of histamine and IL‐4 release. Many in vitro and in vivo studies have demonstrated the anti‐inflammatory and immunomodulatory effects of a supplementation with *M. oleifera* leaves and seeds.^8^ However, occupational asthma induced by *M. oleifera* seed powder has been described, with positive skin‐prick testing and reproducible symptoms on challenge.^9^ We also reported here the third case, to our knowledge, of anaphylactic reaction to *M. oleifera* based on the serum tryptase level and the skin‐prick testing performed with fresh *M. oleifera* leaves, it seems likely that this patient developed IgE sensitization to *M. oleifera* leaves and not to seeds. Indeed, it is possible that the leaves contain specific allergens (not present in seeds) or some cross‐reactive proteins present in other plants, which can be responsible for the anaphylactic reaction. The first case of anaphylaxis to *M. oleifera* was reported in Australia where a 40‐year‐old office‐worker developed widespread angioedema over his face and body within 20 min of ingesting cooked *M. oleifera*, the young seedpod of the Drumstick tree.^10^ The second was reported in France with cooked leaves of *Moringa* in a 30‐year‐old patient treated with anti‐allergic immunotherapy.^11^ Both seeds and leaves were responsible for anaphylactic reactions. These three cases of anaphylactic reactions to *Moringa* known for its anti‐allergic qualities reveal the possible IgE‐mediated hypersensitivity reactions after its consumption.

*M. oleifera* allergy is still a rare condition, but its increased consumption may increase the incidence of *Moring*a‐related allergic reactions. Therefore, we suggest that warning information should be clearly labeled on every preparation containing this plant because our case suggests that *M. oleifera* is a causative allergen in food‐induced anaphylaxis and symptoms of anaphylaxis seen in subjects following ingestion of *M. oleifera* can be true IgE‐mediated hypersensitivity reactions.

## 4 CONCLUSION

This case presents a reminder that although herbal therapies may contain active components with therapeutic efficacy; these plants can also induce aberrant allergic responses, which can be potentially dangerous. Skin‐prick testing on fresh food can be a useful diagnostic tool, as long as adequate positive and negative controls and unaffected healthy controls are in place. Despite reports of *M. oleifera* reducing histamine release from mast cells, a positive skin‐prick test on fresh *M. oleifera* leaves can still be demonstrated in the context of IgE sensitization.

### AUTHOR CONTRIBUTION

Ichrak Bannour conceived, analyzed, drafted, and revised the manuscript.

### CONFLICT OF INTEREST

The author declares no conflicts of interest.

## ETHCIAL APPROVAL

Ethical approval for this case report was not required.

## CONSENT

Written informed consent was obtained from the patient to publish this report in accordance with the journal's patient consent policy.
